# Ethyl 5-bromo­naphtho­[2,1-*b*]furan-2-carboxyl­ate

**DOI:** 10.1107/S160053681205204X

**Published:** 2013-01-09

**Authors:** M. Shet Prakash, P. A. Suchetan, K. M. Mahadevan, V. P. Vaidya, D. Velumurgan, B. S. Palakshamurthy

**Affiliations:** a Department of Studies and Research in Chemistry, U.C.S., Tumkur University, Tumkur, Karnataka 572 103, India; b Department of Chemistry, Kuvempu University, Shankaraghatta Shimoga, Karnataka, India; c Centre of Advanced Study in Crystallography and Biophysics, University of Madras, Guindy Campus, Chennai 600 025, India; d Department of Studies and Research in Physics, U.C.S., Tumkur University, Tumkur, Karnataka 572 103, India

## Abstract

In the title compound, C_15_H_11_BrO_3_, the dihedral angle between the naphtho­furan ring system (r.m.s. deviation = 0.022 Å) and the side chain is 4.50 (2)°. In the crystal, short Br⋯Br [3.4435 (7) Å] contacts propagating along [010] in a zigzag manner and weak π–π inter­actions [shortest centroid–centroid separation = 3.573 (2) Å] directedalong [100] are observed.

## Related literature

For background to the biological activity of naphtho­furan derivatives, see: Vaidya *et al.* (2011 ▶).
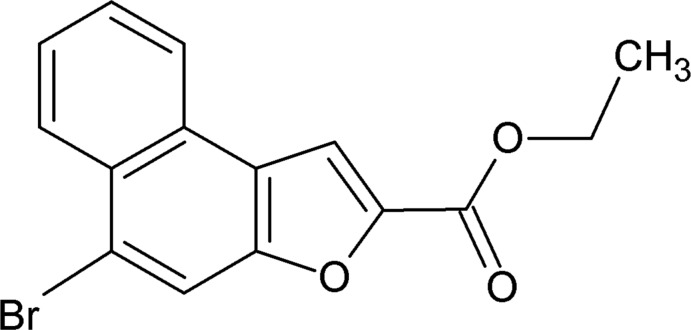


## Experimental

### 

#### Crystal data

C_15_H_11_BrO_3_
*M* *_r_* = 319.15
Monoclinic, 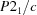

*a* = 7.3108 (4) Å
*b* = 11.1545 (6) Å
*c* = 15.9752 (10) Å
β = 100.921 (4)°
*V* = 1279.16 (13) Å^3^
*Z* = 4
Mo *K*α radiation
μ = 3.21 mm^−1^
*T* = 298 K
0.28 × 0.24 × 0.18 mm

#### Data collection

Bruker APEXII CCD diffractometer
Absorption correction: multi-scan (*SADABS*; Sheldrick, 1996) ▶ *T*_min_ = 0.467, *T*_max_ = 0.595
10028 measured reflections
2249 independent reflections
1699 reflections with *I* > 2σ(*I*)
*R*_int_ = 0.037

#### Refinement

*R*[*F* ^2^ > 2σ(*F* ^2^)] = 0.045
*wR*(*F* ^2^) = 0.127
*S* = 1.08
2249 reflections
172 parameters
H-atom parameters constrained
Δρ_max_ = 0.59 e Å^−3^
Δρ_min_ = −0.54 e Å^−3^

### 

Data collection: *APEX2* (Bruker, 2004 ▶); cell refinement: *SAINT-Plus* (Bruker, 2004 ▶); data reduction: *SAINT-Plus*; program(s) used to solve structure: *SHELXS97* (Sheldrick, 2008 ▶); program(s) used to refine structure: *SHELXL97* (Sheldrick, 2008 ▶); molecular graphics: *ORTEP-3 for Windows* (Farrugia, 2012 ▶); software used to prepare material for publication: *SHELXL97*.

## Supplementary Material

Crystal structure: contains datablock(s) I, global. DOI: 10.1107/S160053681205204X/hb7019sup1.cif

Structure factors: contains datablock(s) I. DOI: 10.1107/S160053681205204X/hb7019Isup2.hkl

Supplementary material file. DOI: 10.1107/S160053681205204X/hb7019Isup3.cml

Additional supplementary materials:  crystallographic information; 3D view; checkCIF report

## Figures and Tables

**Fig. 1.**
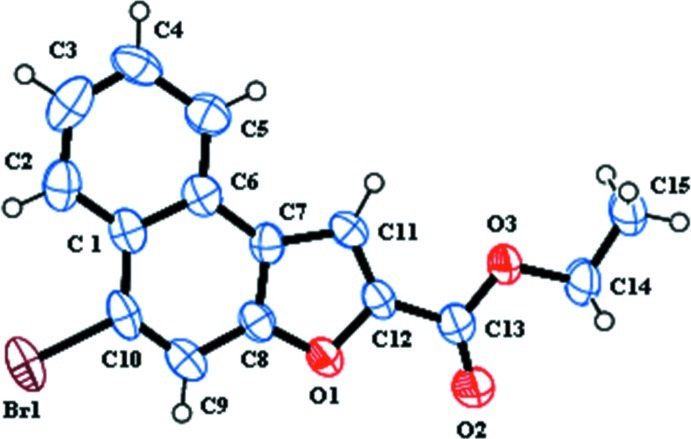
Molecular structure of the title compound, showing displacement ellipsoids drawn at the 50% probability level.

**Fig. 2.**
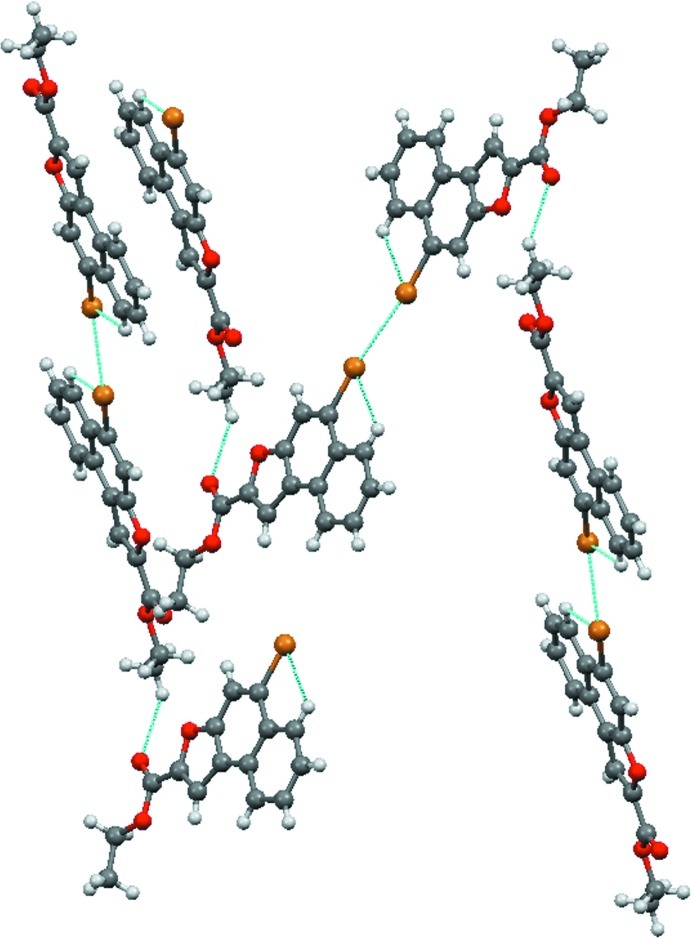
Molecular packing of the title compound. Hydrogen bonds are shown as dashed lines.

**Fig. 3.**
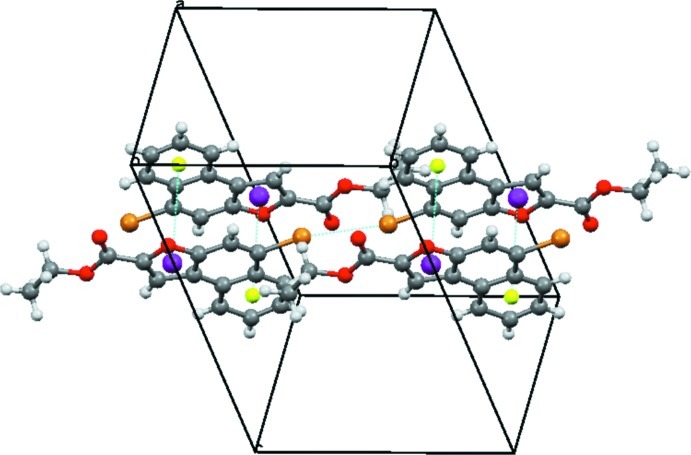
Molecular packing of the title compound through π–π interactions are shown as dashed lines.

## supplementary crystallographic information

### Comment

As part of our ongoing studies of naphthofuran derivatives with possible biological activities (Vaidya *et al.*, 2011), we now describe the structure of the title compound.

The title compound crystallizes in monoclinic crystal system with *P*2_1_/*c* space group. The molecule is essentially planar with the dihedral angle between the mean planes defined by the naphthofuran moiety and the side chain is 4.50 (2)°, and the torsion angle of 179.81 (2)*^o^* for C15—C14—O3—C13 shows that the ethyl group is in planar orientation with the naphthofuran ring. In contrast to this, an antiperiplanar orientation is observed between the ethyl group and the naphthofuran ring in ethylnaphtho[2,1-*b*]furan-2-carboxylate. In the crystal, weak Br···Br and π–π interaction between the rings C1—C6 and O1—C12 occur.

### Experimental

To a solution of ethyl naphtho[2,1-*b*]furan- 2-carboxylate (0.1 mol) in glacial acetic acid (20 ml) was added a solution of bromine (0.1 mol) in acetic acid (20 ml) with stirring during 1 h at 10–20°C and the stirring was continued for 3 h. The reaction mixture was poured into ice-cold water and the solid obtained was filtered out. It was washed with water, dried and the product was recrystallized from ethanol solution as colourless prisms.

### Refinement

The H atoms were positioned with idealized geometry using a riding model with C—H = 0.93 - 0.97 Å. The isotropic displacement parameters for all H atoms were set to 1.2 times of the *U*_eq_ of the parent atom (1.5 times of the *U*_eq_ of the parent atom for CH3).

### Crystal data

**Table:**

C_15_H_11_BrO_3_	prism
*M**_r_* = 319.15	*D*_x_ = 1.657 Mg m^−^^3^
Monoclinic, *P*2_1_/*c*	Melting point: 402 K
Hall symbol: -P 2ybc	Mo *K*α radiation, λ = 0.71073 Å
*a* = 7.3108 (4) Å	Cell parameters from 2249 reflections
*b* = 11.1545 (6) Å	θ = 2.2–25.0°
*c* = 15.9752 (10) Å	µ = 3.21 mm^−^^1^
β = 100.921 (4)°	*T* = 298 K
*V* = 1279.16 (13) Å^3^	Prism, colourless
*Z* = 4	0.28 × 0.24 × 0.18 mm
*F*(000) = 640	

### Data collection

**Table:**

Bruker APEXII CCD diffractometer	2249 independent reflections
Radiation source: fine-focus sealed tube	1699 reflections with *I* > 2σ(*I*)
Graphite monochromator	*R*_int_ = 0.037
Detector resolution: 0.95 pixels mm^-1^	θ_max_ = 25.0°, θ_min_ = 2.2°
phi and ω scans	*h* = −8→8
Absorption correction: multi-scan (*SADABS*; Sheldrick, 1996)	*k* = −12→13
*T*_min_ = 0.467, *T*_max_ = 0.595	*l* = −18→18
10028 measured reflections	

### Refinement

**Table:**

Refinement on *F*^2^	Primary atom site location: structure-invariant direct methods
Least-squares matrix: full	Secondary atom site location: difference Fourier map
*R*[*F*^2^ > 2σ(*F*^2^)] = 0.045	Hydrogen site location: inferred from neighbouring sites
*wR*(*F*^2^) = 0.127	H-atom parameters constrained
*S* = 1.08	*w* = 1/[σ^2^(*F*_o_^2^) + (0.0587*P*)^2^ + 1.4776*P*] where *P* = (*F*_o_^2^ + 2*F*_c_^2^)/3
2249 reflections	(Δ/σ)_max_ < 0.001
172 parameters	Δρ_max_ = 0.59 e Å^−^^3^
0 restraints	Δρ_min_ = −0.54 e Å^−^^3^
0 constraints	

### Special details

**Table:**

Geometry. All e.s.d.'s (except the e.s.d. in the dihedral angle between two l.s. planes) are estimated using the full covariance matrix. The cell e.s.d.'s are taken into account individually in the estimation of e.s.d.'s in distances, angles and torsion angles; correlations between e.s.d.'s in cell parameters are only used when they are defined by crystal symmetry. An approximate (isotropic) treatment of cell e.s.d.'s is used for estimating e.s.d.'s involving l.s. planes.
Refinement. Refinement of *F*^2^ against ALL reflections. The weighted *R*-factor *wR* and goodness of fit *S* are based on *F*^2^, conventional *R*-factors *R* are based on *F*, with *F* set to zero for negative *F*^2^. The threshold expression of *F*^2^ > σ(*F*^2^) is used only for calculating *R*-factors(gt) *etc*. and is not relevant to the choice of reflections for refinement. *R*-factors based on *F*^2^ are statistically about twice as large as those based on *F*, and *R*- factors based on ALL data will be even larger.

### Fractional atomic coordinates and isotropic or equivalent isotropic displacement parameters (Å^2^)

**Table:**

	*x*	*y*	*z*	*U*_iso_*/*U*_eq_	
C15	−0.1065 (7)	−0.5565 (4)	0.3863 (3)	0.0671 (13)	
H15A	−0.1556	−0.6243	0.3523	0.101*	
H15B	−0.1951	−0.5306	0.4198	0.101*	
H15C	0.0077	−0.5787	0.4233	0.101*	
O1	0.1492 (4)	−0.0566 (2)	0.36398 (17)	0.0472 (7)	
C1	0.3625 (5)	0.1416 (3)	0.5778 (3)	0.0429 (10)	
C2	0.4360 (6)	0.2041 (4)	0.6518 (3)	0.0584 (12)	
H2	0.4792	0.2821	0.6487	0.070*	
C3	0.4446 (7)	0.1483 (5)	0.7312 (4)	0.0722 (15)	
H3	0.4956	0.1924	0.7794	0.087*	
C4	0.3872 (6)	0.0395 (4)	0.7436 (3)	0.0581 (12)	
H4	0.3924	0.0076	0.7978	0.070*	
C5	0.3127 (6)	−0.0296 (4)	0.6643 (3)	0.0552 (11)	
H5	0.2734	−0.1083	0.6687	0.066*	
C6	0.3009 (5)	0.0217 (3)	0.5838 (3)	0.0442 (9)	
C7	0.2283 (5)	−0.0402 (3)	0.5065 (3)	0.0402 (9)	
C8	0.2182 (5)	0.0167 (3)	0.4302 (3)	0.0420 (9)	
C9	0.2729 (6)	0.1358 (3)	0.4210 (3)	0.0497 (10)	
H9	0.2609	0.1722	0.3678	0.060*	
C10	0.3435 (5)	0.1938 (3)	0.4934 (3)	0.0487 (11)	
C11	0.1573 (5)	−0.1595 (3)	0.4862 (3)	0.0422 (9)	
H11	0.1442	−0.2204	0.5244	0.051*	
C12	0.1141 (5)	−0.1642 (3)	0.4006 (3)	0.0421 (9)	
C13	0.0419 (6)	−0.2605 (4)	0.3405 (3)	0.0488 (10)	
C14	−0.0706 (7)	−0.4583 (4)	0.3302 (3)	0.0616 (12)	
H14A	0.0183	−0.4838	0.2958	0.074*	
H14B	−0.1852	−0.4355	0.2923	0.074*	
O2	0.0212 (6)	−0.2527 (3)	0.2649 (2)	0.0744 (10)	
O3	0.0034 (4)	−0.3571 (2)	0.38317 (19)	0.0535 (8)	
Br1	0.42767 (7)	0.35357 (4)	0.48343 (4)	0.0708 (2)	

### Atomic displacement parameters (Å^2^)

**Table:**

	*U*^11^	*U*^22^	*U*^33^	*U*^12^	*U*^13^	*U*^23^
C15	0.088 (3)	0.045 (3)	0.069 (3)	−0.018 (2)	0.017 (3)	−0.009 (2)
O1	0.0569 (16)	0.0351 (15)	0.0501 (17)	−0.0048 (12)	0.0113 (13)	0.0043 (12)
C1	0.0341 (18)	0.034 (2)	0.063 (3)	0.0046 (16)	0.0143 (18)	−0.0021 (19)
C2	0.053 (3)	0.045 (3)	0.075 (3)	0.000 (2)	0.007 (2)	−0.014 (2)
C3	0.064 (3)	0.082 (4)	0.065 (4)	0.009 (3)	−0.003 (3)	−0.029 (3)
C4	0.046 (2)	0.059 (3)	0.069 (3)	0.007 (2)	0.009 (2)	0.030 (2)
C5	0.057 (2)	0.051 (3)	0.059 (3)	0.006 (2)	0.014 (2)	0.007 (2)
C6	0.0378 (19)	0.040 (2)	0.056 (3)	0.0072 (16)	0.0116 (18)	0.0010 (19)
C7	0.0356 (18)	0.033 (2)	0.052 (2)	0.0031 (15)	0.0074 (17)	−0.0044 (18)
C8	0.0411 (19)	0.034 (2)	0.053 (3)	0.0019 (16)	0.0152 (18)	0.0009 (19)
C9	0.053 (2)	0.037 (2)	0.063 (3)	0.0029 (18)	0.020 (2)	0.008 (2)
C10	0.040 (2)	0.0272 (19)	0.081 (3)	−0.0003 (16)	0.018 (2)	−0.001 (2)
C11	0.044 (2)	0.035 (2)	0.049 (3)	0.0037 (16)	0.0113 (18)	0.0072 (18)
C12	0.042 (2)	0.030 (2)	0.054 (3)	−0.0015 (16)	0.0098 (18)	0.0041 (17)
C13	0.051 (2)	0.044 (2)	0.052 (3)	−0.0052 (18)	0.010 (2)	0.000 (2)
C14	0.083 (3)	0.045 (2)	0.057 (3)	−0.015 (2)	0.014 (2)	−0.015 (2)
O2	0.114 (3)	0.063 (2)	0.046 (2)	−0.027 (2)	0.0132 (19)	−0.0010 (16)
O3	0.0742 (19)	0.0390 (16)	0.0463 (17)	−0.0151 (14)	0.0087 (15)	−0.0038 (13)
Br1	0.0814 (4)	0.0354 (3)	0.0987 (5)	−0.0111 (2)	0.0247 (3)	0.0021 (2)

### Geometric parameters (Å, º)

**Table:**

C15—C14	1.470 (7)	C6—C7	1.426 (6)
C15—H15A	0.9600	C7—C8	1.363 (6)
C15—H15B	0.9600	C7—C11	1.443 (5)
C15—H15C	0.9600	C8—C9	1.403 (5)
O1—C8	1.357 (5)	C9—C10	1.341 (6)
O1—C12	1.380 (4)	C9—H9	0.9300
C1—C2	1.389 (6)	C10—Br1	1.902 (4)
C1—C6	1.420 (5)	C10—Br1	1.902 (4)
C1—C10	1.451 (6)	C11—C12	1.345 (6)
C2—C3	1.404 (8)	C11—H11	0.9300
C2—H2	0.9300	C12—C13	1.471 (6)
C3—C4	1.311 (7)	C13—O2	1.192 (5)
C3—H3	0.9300	C13—O3	1.333 (5)
C4—C5	1.495 (7)	C14—O3	1.452 (5)
C4—H4	0.9300	C14—H14A	0.9700
C5—C6	1.396 (6)	C14—H14B	0.9700
C5—H5	0.9300	Br1—Br1	0.0000
			
C14—C15—H15A	109.5	O1—C8—C9	124.0 (4)
C14—C15—H15B	109.5	C7—C8—C9	124.5 (4)
H15A—C15—H15B	109.5	C10—C9—C8	115.9 (4)
C14—C15—H15C	109.5	C10—C9—H9	122.1
H15A—C15—H15C	109.5	C8—C9—H9	122.1
H15B—C15—H15C	109.5	C9—C10—C1	124.2 (4)
C8—O1—C12	105.4 (3)	C9—C10—Br1	117.2 (3)
C2—C1—C6	119.5 (4)	C1—C10—Br1	118.5 (3)
C2—C1—C10	122.9 (4)	C9—C10—Br1	117.2 (3)
C6—C1—C10	117.6 (4)	C1—C10—Br1	118.5 (3)
C1—C2—C3	119.4 (4)	Br1—C10—Br1	0.00 (4)
C1—C2—H2	120.3	C12—C11—C7	105.7 (3)
C3—C2—H2	120.3	C12—C11—H11	127.2
C4—C3—C2	125.9 (5)	C7—C11—H11	127.2
C4—C3—H3	117.1	C11—C12—O1	111.7 (3)
C2—C3—H3	117.1	C11—C12—C13	132.7 (4)
C3—C4—C5	115.1 (5)	O1—C12—C13	115.6 (4)
C3—C4—H4	122.4	O2—C13—O3	125.4 (4)
C5—C4—H4	122.4	O2—C13—C12	124.7 (4)
C6—C5—C4	121.2 (4)	O3—C13—C12	110.0 (4)
C6—C5—H5	119.4	O3—C14—C15	108.3 (4)
C4—C5—H5	119.4	O3—C14—H14A	110.0
C5—C6—C1	118.9 (4)	C15—C14—H14A	110.0
C5—C6—C7	123.1 (4)	O3—C14—H14B	110.0
C1—C6—C7	118.0 (4)	C15—C14—H14B	110.0
C8—C7—C6	119.8 (4)	H14A—C14—H14B	108.4
C8—C7—C11	105.8 (4)	C13—O3—C14	114.9 (3)
C6—C7—C11	134.4 (4)	Br1—Br1—C10	0
O1—C8—C7	111.4 (3)		
